# Supplementary material for: PARM1 Drives Smooth Muscle Cell Proliferation in Pulmonary Arterial Hypertension via AKT/FOXO3A Axis
Source: Int J Mol Sci. 2023 Mar 28;24(7):6385. doi: 10.3390/ijms24076385 (PMC10094810; doi:10.3390/ijms24076385)
Supplement: Supplementary file 1 [file ijms-24-06385-s001.zip › ijms-2072878-supplementary.pdf]

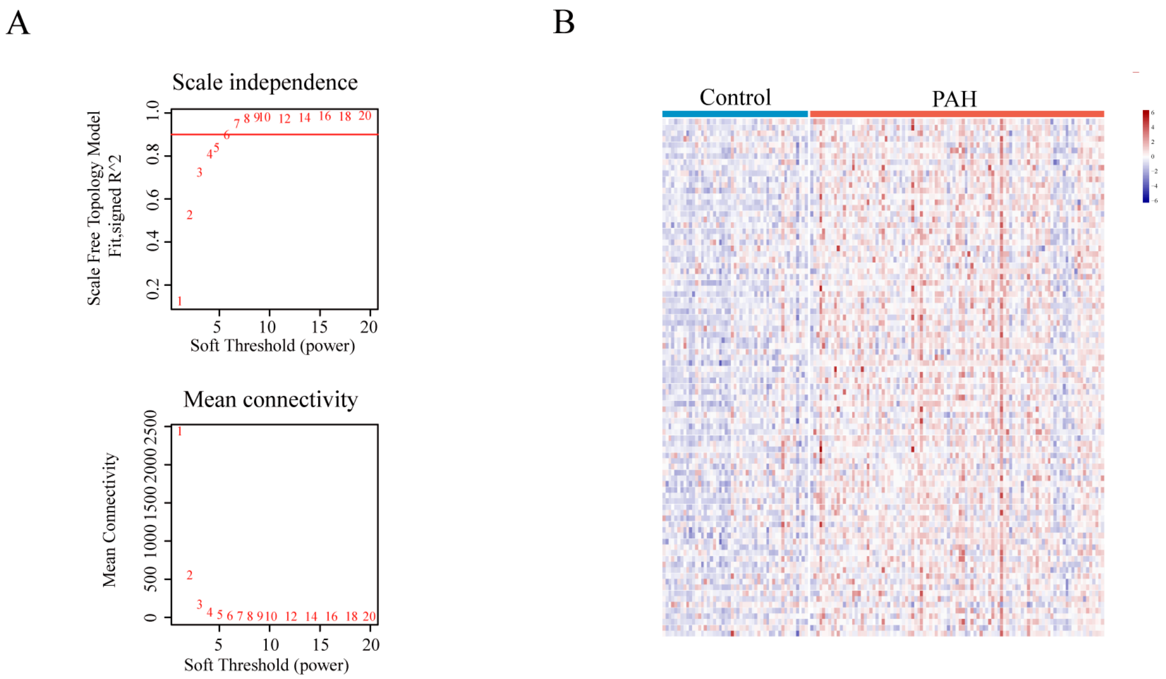

Figure S1. The module detection by WGCNA. (A) In the scale-free topology plot, the up panel shows the scale-free index as a function of the soft thresholding power. The down panel displays the mean connectivity as a function of the soft-thresholding power. (B) The heat map of the white gene expression between normal lungs and PAH lungs. WGCNA, weighted gene co-expression network analysis; PAH, pulmonary arterial hypertension.

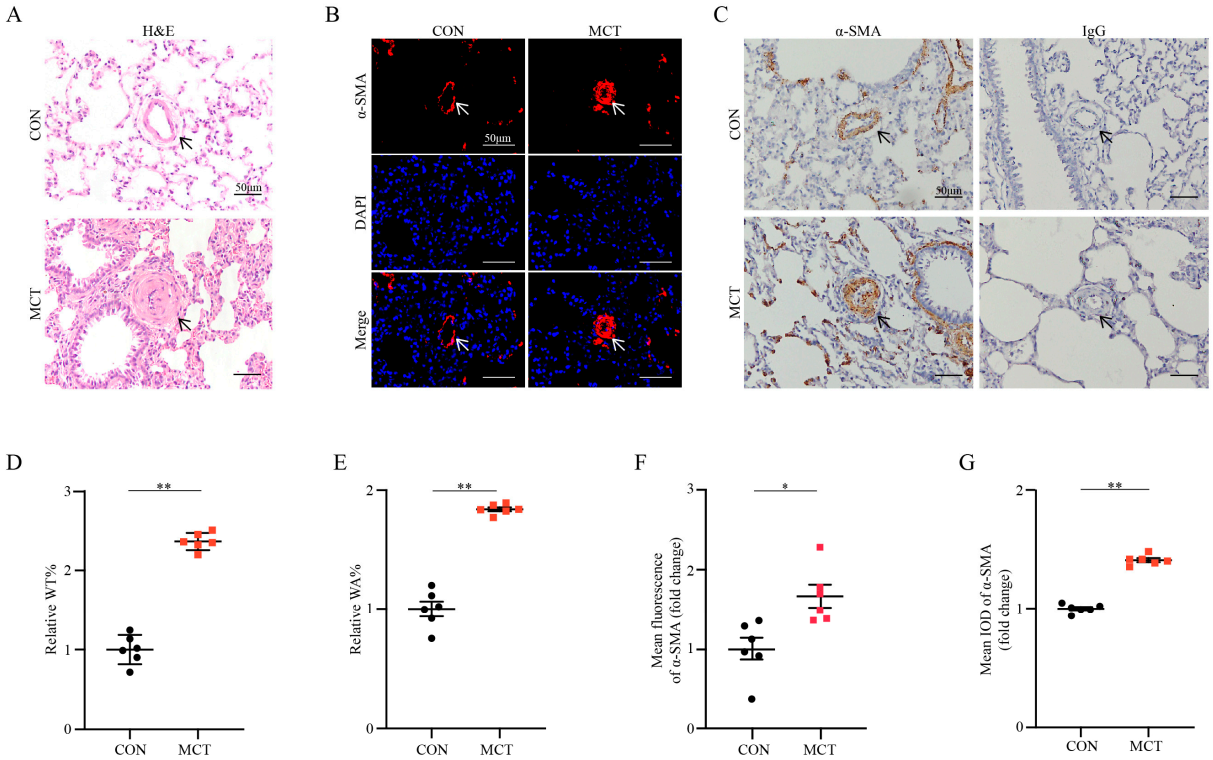

Figure S2. H&E staining and immunohistochemical staining of rat pulmonary artery. (A) Representative images of H&E staining in the pulmonary artery of CON and PAH rats (n=6; scale bar, 50 μm). (B) Representative images of immunofluorescence staining in the pulmonary artery of CON and PAH rats (n=6; scale bar, 50 μm). (C) Representative images of immunohistochemical staining in the pulmonary artery of CON and PAH rats (n=6; scale bar, 50 μm). (D, E) Quantitative analysis of WT% and WA% of pulmonary artery of H&E staining (n=6 each). (F) Representative immunofluorescence staining quantification of α-SMA. (G) Representative immunohistochemical quantification of α-SMA. Data are shown as mean ± SEM. *$p < 0.05$, **$p < 0.01$ vs. CON group (student's t-test). CON, control rats without pulmonary arterial hypertension; PAH, pulmonary arterial hypertension; MCT, monocrotaline; H&E, hematoxylin eosin; WT%, (the diameter of external vessel – the diameter of internal vessel) / the diameter of external vessel × 100%; WA%, (the total area of the vessel – the lumen area of the vessel) / the total area of vessel × 100%; IOD, the integral optical density; α-SMA, α-smooth muscle actin. The black/white arrow represents pulmonary artery.

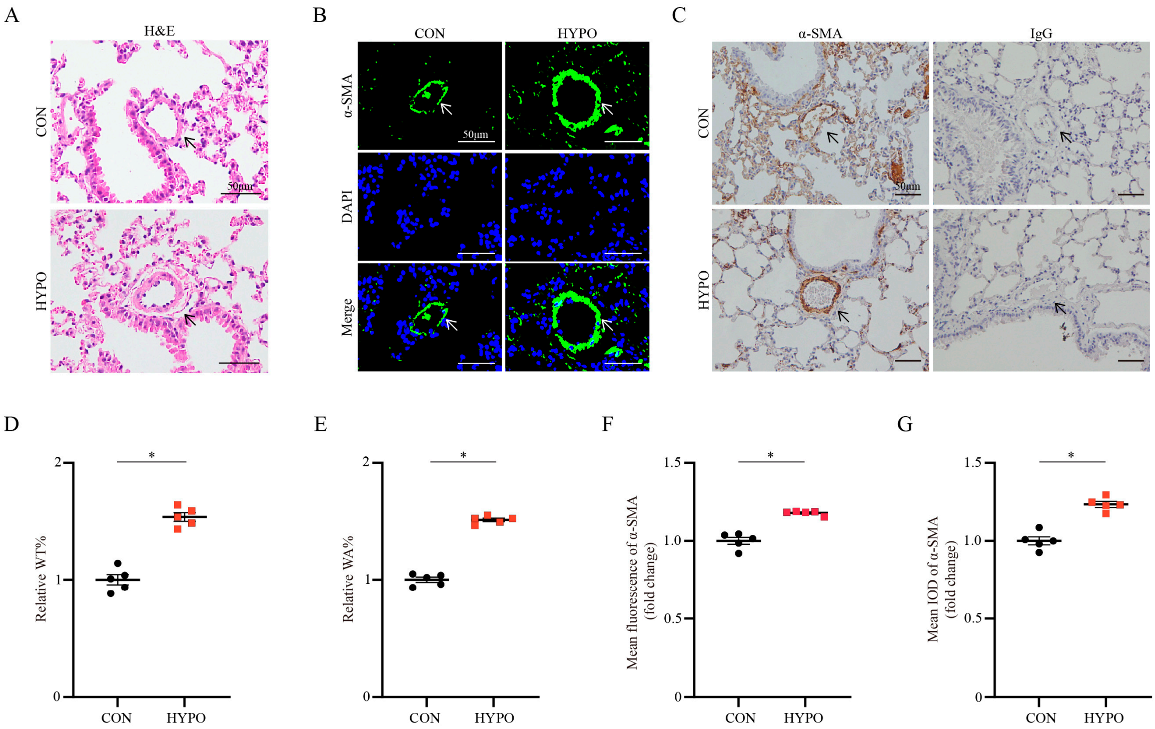

Figure S3. H&E staining and immunohistochemical staining of mouse pulmonary artery. (A) Representative images of H&E staining in the pulmonary artery of CON and PAH mice (n=5; scale bar, 50 μm). (B) Representative images of immunofluorescence staining in the pulmonary artery of CON and PAH mice (n=5; scale bar, 50 μm). (C) Representative images of immunohistochemical staining in the pulmonary artery of CON and PAH mice (n=5; scale bar, 50 μm). (D, E) Quantitative analysis of WT% and WA% of pulmonary artery of H&E staining (n=5 each). (F) Representative immunofluorescence staining quantification of α-SMA. (G) Representative immunohistochemical quantification of α-SMA. Data are shown as mean ± SEM. **p* < 0.05 vs. CON group (student t-test). CON, control mice without pulmonary arterial hypertension; PAH, pulmonary arterial hypertension; HYPO, hypoxia; H&E, hematoxylin eosin; WT%, (the diameter of external vessel – the diameter of internal vessel) / the diameter of external vessel × 100%; WA%, (the total area of the vessel – the lumen area of the vessel) / the total area of vessel × 100%; IOD, the integral optical density; α-SMA, α-smooth muscle actin. The black/white arrow represents pulmonary artery.

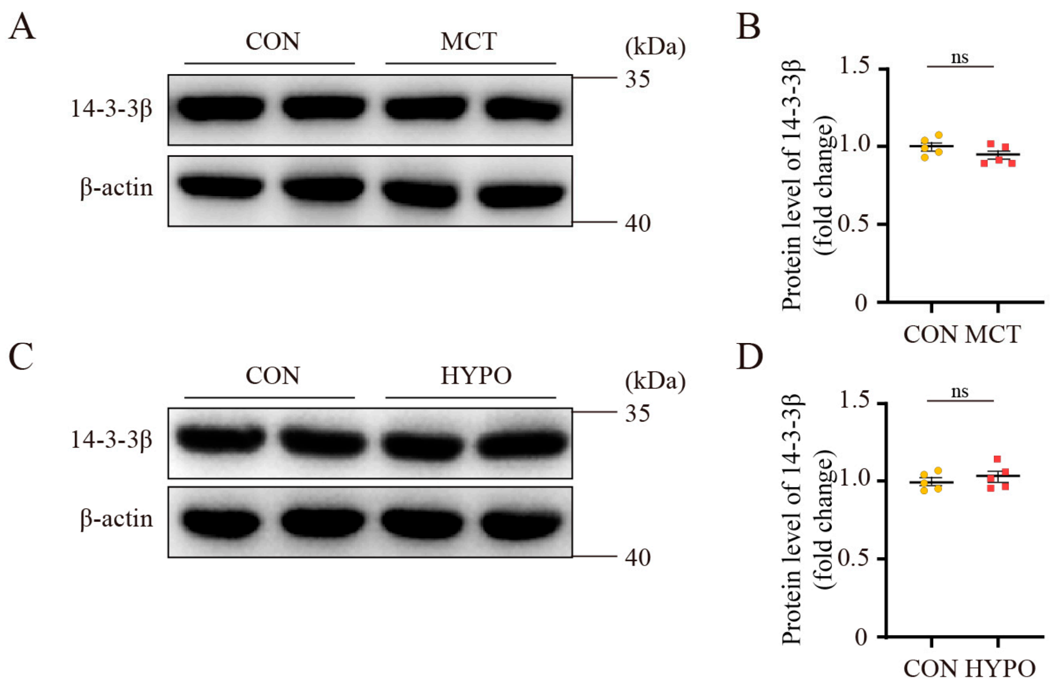

Figure S4. Representative Western blots and analysis of 14-3-3β expression in rat/mouse lung. (A, B) Representative images of Western blots of 14-3-3β expression in rat lung. (C, D) Representative images of Western blots of 14-3-3β expression in mouse lung.Equal protein loading was confirmed using an anti-β-actin antibody (n=5 each). Data are shown as mean ± SEM (n=5 each); ns represents statically non-significant (student's *t*-test).

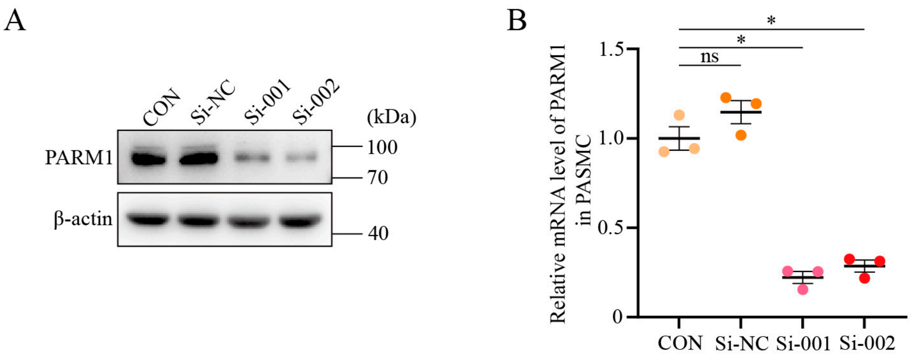

Figure S5. Validation of PARM1 siRNAs in PASMC. Different siRNA of PARM1, Si-001 and Si-002, as well as scrambled control, Si-NC were tested in primarily isolated PASMC. Representative Western blots (A) and qPCR results as summarized in a dot plot (B) showed that both siRNAs could significantly downregulate the expression of PARM1 in PASMC. Data are shown as mean ± SEM (n=3); ns represents statically non-significant. *$p < 0.05$ vs. CON group (student's t-test).
